# Exonic variants of genes related to the vitamin D signaling pathway in the families of familial multiple sclerosis using whole‐exome next generation sequencing

**DOI:** 10.1002/brb3.1272

**Published:** 2019-03-21

**Authors:** Vanesa Pytel, Jordi A. Matías‐Guiu, Laura Torre‐Fuentes, Paloma Montero‐Escribano, Paolo Maietta, Javier Botet, Sara Álvarez, Ulises Gómez‐Pinedo, Jorge Matías‐Guiu

**Affiliations:** ^1^ Department of Neurology, Institute of Neurosciences, IdISSC, Hospital Clínico San Carlos Universidad Complutense de Madrid Madrid Spain; ^2^ Laboratory of Neurobiology, Institute of Neurosciences, IdISSC, Hospital Clínico San Carlos Universidad Complutense de Madrid Madrid Spain; ^3^ Nimgenetics Madrid Spain

**Keywords:** cubilin, *CYP24A1*, familial multiple sclerosis, megalin, *PDIA3*, *VDR*, vitamin D, whole‐exome sequencing

## Abstract

**Introduction:**

Vitamin D (VD) deficiency has been associated with multiple sclerosis (MS) and other autoimmune diseases (AIDs). However, the effect of the genetics of VD on the risk of MS is subject to debate. This study focuses on genes linked to the VD signaling pathway in families with MS. The evaluation of gene variants in all the members of families could contribute to an additional knowledge on the information obtained from case‐control studies that use nonrelated healthy people.

**Material and Methods:**

We studied 94 individuals from 15 families including at least two patients with MS. We performed whole‐exome next generation sequencing on all individuals and analyzed variants of the *DHCR7*, *CYP2R1*, *CYP3A4*, *CYP27A1*, *GC*, *CYP27B1*, *LRP2*, *CUBN*, *DAB2*, *FCGR*, *RXR*, *VDR*, *CYP24A1,* and *PDIA3* genes. We also studied *PTH*, *FGF23*, *METTL1*, *METTL21B,* and the role of the linkage disequilibrium block on the long arm of chromosome 12, through analysis of the *CDK4*, *TSFM*, *AGAP2*, and *AVIL* genes. We compared patients with MS, other AIDs and unaffected members from different family types.

**Results:**

The study described the variants in the VD signaling pathway that appear in families with at least two patients with MS. Some infrequent variants were detected in these families, but no significant difference was observed between patients with MS and/or other AIDs and unaffected family members in the frequency of these variants. Variants previously associated with MS in the literature were not observed in these families or were distributed similarly in patients and unaffected family members.

**Conclusion:**

The study of genes involved in the VD signaling pathway in families that include more than one patient with MS did not identify any variants that could explain the presence of the disease, suggesting that VD metabolism could probably play a role in MS more as an environmental factor rather than as a genetic factor. Our study also supports the analysis of cases and unaffected individuals within families in order to determine the influence of genetic factors.

**Abbreviations**
1,25(OH)2D: 1,25‐dihydroxyvitamin D
1,25(OH)2D3: 1,25‐dihydroxyvitamin D3
7‐DHC: 7 dehydrocholesterol
25(OH)D: 25‐hydroxyvitamin D3
AID: autoimmune disease
CCDS: consensus coding sequence
CNS: central nervous system
DBP: vitamin D binding protein
FGF23: phosphaturic factor fibroblast growth factor 23
GWAS: genome‐wide association study
LD: linkage disequilibrium
MAF: minor allele frequency
MS: multiple sclerosis
PDIA3: Protein disulfide isomerase family member 3
PPMS: primary‐progressive multiple sclerosis
PTH: parathyroid hormone
RRMS: relapsing‐remitting multiple sclerosis
RXRA: retinoid X receptor alpha
SPMS: secondary‐progressive multiple sclerosis
VD: vitamin D
VDR: vitamin D receptor
WES: whole‐exome next generation sequencing

## 1 INTRODUCTION

Multiple sclerosis (MS) is an inflammatory autoimmune disease of the central nervous system (CNS) that causes demyelination and axonal damage. MS etiology involves multiple factors, with environmental factors interacting with genetic predisposition. Although genome‐wide association studies (GWAS) have validated the central role of the major histocompatibility complex in the genetics of MS, susceptibility has been found to be influenced by several genes outside the short arm of chromosome 6 (Baranzini & Oksenberg, 2017; Cree, 2014; Sawcer et al., 2011). The disease is therefore considered to be polygenic. However, the variants identified through GWAS have little effect on the overall risk of MS; as a result, there is a considerable need for further research into genetic factors in MS. New techniques such as whole‐exome sequencing (WES) offer a comprehensive view of the coding region of the genome, allowing us to study complex diseases like MS with more detail.

**Figure 1:**
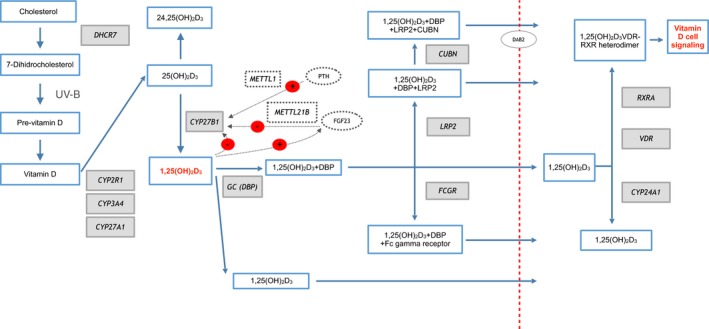
Vitamin D pathway. 7‐DHC follows a two‐stage reaction involving ultraviolet‐B irradiation and thermal isomerization. Vitamin D is converted into 25‐hydroxyvitamin D3 (25[OH]D_3_) by a hydroxylation mechanism mediated by vitamin D 25 hydroxylase. Subsequently, 1α‐hydroxylase, regulated by PTH and FGF23, transforms 25(OH)D_3_ into its active form, 1,25‐dihydroxyvitamin D3 (1,25[OH]_2_D_3_). The proteins 25(OH)D_3_ and 1,25 (OH)_2_D_3 _mainly circulate bound to carrier proteins (DBP), but a small fraction circulates freely or bound to albumin. The internalization mechanism is produced mainly by DBP‐megalin (*LRP2*). However, in some cells this process can occur by binding to other proteins and even by passive diffusion. Finally, 1,25(OH)_2_D_3_binds to the VDR nuclear receptor, interacting with the RXRA, forming a heterodimer that allows transport to the cell nucleus. Genes involved in the vitamin D pathway are shown in grey. The regulatory pathway of the gene *CYP27B1 *(*METTL1*,* METTL21B*,* FGF23*,* PTH*) is represented with a dotted line. Endocytosis of the complex formed by LRP2 and CUBN requires DAB2, represented in the figure within a light grey circle. The mechanism of VD binding to the PDIA3 receptor is not included in the figure

Vitamin D (VD) is a fat‐soluble hormone which plays an essential role in calcium homeostasis and in skeletal development and maintenance. It is also thought to play a role in cancer, immune function, and autoimmune diseases (AIDs), including MS (Ascherio et al., 2014; Gianfrancesco et al., 2017; Løken‐Amsrud et al., 2012; Mowry et al., 2012; Munger & Ascherio, 2011; Munger, Levin, Hollis, Howard, & Ascherio, 2006; Salzer et al., 2012; Simpson et al., 2010; Wang, Zeng, Wang, & Guo, 2018). There are several types of VD, including ergocalciferol (D_2_) and cholecalciferol (D_3_), formed from their respective previtamins, ergosterol, and 7‐dehydrocholesterol (7‐DHC). The main natural source of VD_3_ in humans is production in the skin, where 7‐DHC undergoes a 2‐step reaction involving ultraviolet‐B irradiation to form pre‐VD_3_, followed by thermal isomerization, forming VD_3_. Both VD_2_ and VD_3_ can also be obtained in small amounts from a varied diet, and in larger amounts from specific foods or nutritional supplements. Dietary VD is mainly absorbed in the small intestine by means of chylomicrons, which enter the lymphatic system and drain into the superior vena cava. After entering the bloodstream, VD (obtained either through intestinal absorption or synthesis in the skin) is converted into 25‐hydroxyvitamin D (25[OH]D) in the liver, and then into 1,25‐dihydroxyvitamin D (1,25[OH]_2_D). Both compounds are mainly transported by vitamin D‐binding protein (DBP, encoded by the *GC* gene), although a small fraction circulates freely or bound to albumin. Internalization is mainly mediated by the DBP‐megalin (*LRP2*) complex. In some cells, however, internalization may take place through binding to other proteins or by passive diffusion of free molecules (Figure 1). It is subsequently converted to 25‐hydroxyvitamin D_3_ (25[OH]D_3_, calcifediol) by cytochrome P450 2R1 (or vitamin D 25‐hydroxylase, encoded by *CYP2R1*). Another cytochrome P450 enzyme, 1α‐hydroxylase (*CYP27B1*), converts this to the active form, 1,25‐dihydroxyvitamin D_3_ (1,25[OH]_2_D_3_, calcitriol)_._ This step appears to be regulated by parathyroid hormone (PTH) and phosphaturic factor fibroblast growth factor 23 (FGF23) (Christakos, 2017). It has also been suggested that this may in turn be influenced by intronic variants located in the nearby genes *METTL1* and *METTL21B* (methyltransferase‐like proteins). CYP24A1 may convert 25(OH)D_3_ into the inactive metabolite 24,25(OH)_2_D_3_, or even convert the active form of the vitamin into an inactive form, 1,24,25(OH)_2_D_3_. 1,25[OH]_2_D_3_ binds to the nuclear vitamin D receptor (VDR), interacting with retinoid X receptor alpha (RXRA) to form a heterodimer, enabling transport into the cell nucleus (Matías‐Guiu, Oreja‐Guevara, Matías‐Guiu, & Gomez‐Pinedo, 2018) (Figure 1). 1,25 [OH] 2D3 can also bind to the membrane receptor PDIA3 (*ERp57* or *1,25 D3‐MARRS*) that could be responsible for the actions of nongenomic mechanisms of VD.

Low‐frequency genetic variants in VD signaling pathway have been linked to MS; however, their role in disease pathogenesis is controversial. We are yet to determine their influence over familial forms of MS, whether the association is due to variations in a gene, or whether the presence of variants at different points in the pathway may be associated with the disease.

The aim of the present study is to analyze the genetic variations of genes related with the VD signaling pathway in families from familial forms of MS. By studying families, genetic factors may be more easily observable and less obscured by environmental factors than in sporadic cases.

## 2 MATERIAL AND METHODS

### 2.1 Study population

We studied 15 families with at least two patients with MS according to the McDonald criteria (Polman et al., 2011), in total 94 individuals. The study protocol included a specifically designed questionnaire to gather information about patients’ personal and family histories of neurological, systemic, and autoimmune conditions. A modified version of the list of diseases created by the American Autoimmune Related Diseases Association (American Autoimmune Related Diseases Association), retrieved from http://www.aarda.org/autoimmune-information/list-of-diseases/) (February, 2016), was used to define the history of AIDs. Cases with no history of MS or other AIDs were considered unaffected members, and data was obtained through direct interrogation. We also recorded demographic and clinical variables, sex, age at onset, time since symptom onset, and clinical form (relapsing‐remitting MS [RRMS], primary‐progressive MS [PPMS], or secondary‐progressive MS [SPMS]). We gathered information on family members by interviewing them directly; families were classified according to the types of family (A or B) defined in a previous study (Pytel et al., 2017): Type A, in which all members with MS belonged to the same generation; and type B, in which MS patients were distributed between two or more generations. The software *Genial Pedigree Draw* (http://app.pedigreedraw.com) was used to generate pedigrees for each family included. We also analyzed the duration of daylight for the habitual place of residence for each individual studied, in order to evaluate the impact of sunlight exposure on the risk of MS; the Spanish National Geographic Institute (www.ign.es) was used as the source for this analysis. Finally, we performed WES studies of peripheral blood samples from the 94 participants.

### 2.2 Whole‐exome sequencing

DNA was extracted from blood samples using the MagNA Pure automated nucleic acid purification system (*Roche Molecular Systems, Inc*.). The Qubit™ 2.0 and NanoDrop devices (Thermo Fisher Scientific Inc.) were used to determine DNA concentration and purity. The AmpliSeq™ Exome panel (Thermo Fisher Scientific Inc.) was used for library preparation. This technique captures >97% of consensus coding sequences (>19,000 genes, >198,000 exons, >85% of alterations responsible for genetic diseases) and adjacent splicing regions (5 bp). The panel is approximately 33 Mb in size and comprises a total of 293,903 amplicons. Libraries were quantified by qPCR and subsequently prepared and enriched using the Ion Chef™ system providing a high uniformity of coverage.

Library sequencing at a mean depth of coverage of >100 reads was performed using the Ion Proton (Thermo Fisher Scientific Inc.) sequencing platform, covering >90% of amplicons with at least 20 reads. The sequences obtained were aligned against the reference genome (Genome Reference Consortium human genome 19, build 37) using the Torrent Mapping Alignment Program software. The sequences, aligned and filtered according to specific quality criteria, were analyzed with the Torrent Variant Caller tool to identify nucleotide variations with respect to the reference genome. Variant annotation was performed using the latest available version of Ion Reporter™ (Thermo Fisher Scientific Inc.). Our analysis aimed to identify SNPs and indels located in the exons and splicing junctions of the genes and causing protein‐level modifications (excluding synonymous variants), which were detected in over 40% of reads.

### 2.3 Variant prioritization

Observing quality controls and bioinformatic filtering, we analyzed the coding and splicing regions of genes involved in the VD signaling pathway. We evaluated the list of variants identified against database information on previously described variants (http://www.ncbi.nlm.nih.gov/SNP/, http://www.1000genomes.org, http://gnomad.broadinstitute.org, and http://evs.gs.washington.edu/EVS). Firstly, we excluded variants located in introns, intergenic regions, and untranslated regions. We also removed synonymous variants; according to a polygenic pattern, we considered variants with a minor allele frequency (MAF) below 5% (http://gnomad.broadinstitute.org/). In order to understand the possible biological functions of the variants selected, we estimated the functional effect of the genomic variations classified as pathogenic using seven prediction algorithms (SIFT, PROVEAN, PolyPhen2, Mutation Taster, Mutation Assessor, LRT, and FATHMM) included in the ALAMUT (http://www.interactive-biosoftware.com) and ANNOVAR (http://www.openbioinformatics.org/annovar/) analysis packages. Finally, we reviewed candidate genes in publications on PubMed and the Online Mendelian Inheritance in Man database.

### 2.4 Analysis of vitamin D signaling pathways

In order to analyze the exonic variants detected, we defined that VD signaling pathway includes the following genes: *DHCR7*, *CYP2R1*, *CYP3A4*, *CYP27A1*, *GC*,* LRP2*,* CUBN*,* FCGR*,* DAB2*,* PTH*, *FGF23*,* METTL1*, *METTL21B CYP27B1*, *RXRA*, *CYP24A1*, *VDR *and *PDIA3* (Kamisli et al., 2018; Tajouri et al., 2005). Given the existence of a linkage disequilibrium (LD) block on the long arm of chromosome 12, centered on the *CYP27B1*, *METTL1*, and *METTL21B *genes, we also analyzed some genes in this area that may be related to autoimmunity (*CDK4*, *TSFM*, *AGAP2*, *AVIL*, *CYP27B1*, *METTL1*, and *METT21B*).

### 2.5 Sequencing data analysis

Sequencing results were evaluated using three analysis models:

1. Comparison of the variants detected in the overall cohort of family members, analysing the groups of individuals with MS, other AIDs, and, unaffected family members, taking into account the type of familial MS.
2. Prioritization of variants.
3. Analysis of pedigrees, assessing the role of the variants identified in each pedigree.

### 2.6 Statistical analysis

Descriptive analysis results are expressed as absolute frequencies and percentages (n [%]), means ± standard deviation (*SD*), or medians (interquartile range). The Kolmogorov‐Smirnov test was used to test quantitative data for normal distribution. The chi‐square test was used to compare independent samples with qualitative variables; the Mann‐Whitney U test was used for quantitative variables. Intergroup differences were evaluated with the Kruskal‐Wallis H test and the Dunn post hoc test. More powerful quasi‐likelihood score test (M_QLS_) was used, which allows testing for case‐control associations in samples with related individuals (Thornton & McPeek, 2007; Thornton, Zhang, Cai, Ober, & McPeek, 2012). Allele frequencies were tested to identify deviations from Hardy‐Weinberg equilibrium. Bonferroni method was used to correct for multiple comparisons. Statistical significance was set at *p* < 0.05.

## 3 RESULTS

### 3.1 Description of the families, population, and daylight exposition

**Figure 2:**
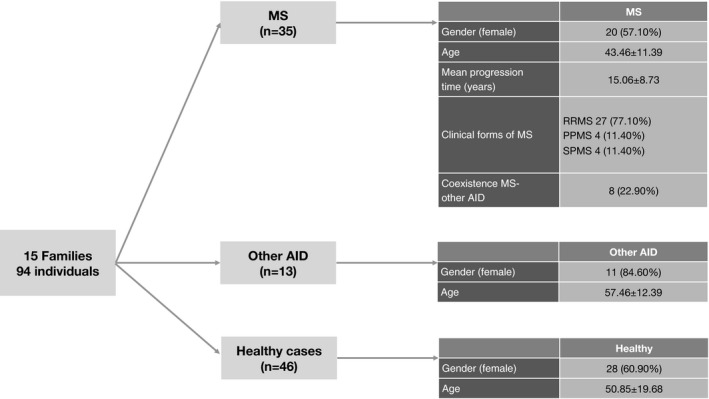
Demographic and clinical characteristics of the study population (*n* = 94; 15 families). RRMS, relapsing‐remitting multiple sclerosis; PPMS, primary‐progressive multiple sclerosis; SPMS, secondary‐progressive multiple sclerosis

We defined two types of family: type A and type B. The sample included seven type‐A families (44 individuals, 46.8%) and eight type‐B families (50; 53.2%). Fifteen patients with MS (42.8%) belonged to type‐A families and 20 (57.14%) belonged to type‐B families. Seven individuals with other AIDs (53.8%) and 22 unaffected individuals (47.8%) belonged to type‐A families and six individuals with other AIDs (46.1%) and 24 unaffected individuals (52.1%) belonged to type‐B families. Of the 94 individuals studied, 35 were diagnosed with MS, 46 had no AIDs, and 13 had other AIDs than MS (hypothyroidism in seven, hyperthyroidism in one, rheumatoid arthritis in two, type 1 diabetes mellitus in one, autoimmune uveitis in one, and systemic lupus erythematosus in one). Of the 35 patients with MS, eight had an additional AID (hypothyroidism in one, type 1 diabetes mellitus in one, ulcerative colitis in one, autoimmune uveitis in one, Guillain‐Barré syndrome in one, systemic lupus erythematosus in one, and rheumatic fever in one). Figure 2 shows the demographic and clinical characteristics of the study population, including the clinical form of MS. Regarding daylight duration (Villar‐Quiles et al., 2016), 91 individuals (96.8%) lived in Madrid from at least 2016, one (1.06%) lived in Miami (USA), one lived in Seville, and one lived in Toledo. The cumulative daylight duration for 2016 was 4,468.4 hr in Madrid, 4,447.8 hr in Miami, 4,459.8 hr in Seville, and 4,463.3 hr in Toledo. By group, 33 patients with MS lived in Madrid, one lived in Miami, and one lived in Toledo. The mean daylight duration for the MS group was 4,467.6 ± 3.5 hr. All family members with other AIDs lived in Madrid, with 4,468.4 ± 0.0 hr of daylight. Of the unaffected family members, one lived in Seville and the rest lived in Madrid, with 4,468.2 ± 1.2 hr of daylight.

### 3.2 Exonic variants of genes involved in the vitamin D signaling pathway

Analysis of the genes associated with the VD signaling pathway revealed a total of 154 different variants in the 94 individuals tested. Disregarding synonymous variants gives a final total of 77 different nonsynonymous variants (Table 1). All variants found followed the Hardy‐Weinberg equilibrium.

**Table 1:** Nonsynonymous exonic variants detected in a cohort of 94 individuals belonging to 15 families including at least two patients with multiple sclerosis

Gene	Locus	Variant (rs)	MAF	Variant effect	Reference allele	Change	MS (*n* = 35)	Other AID (*n* = 13)	Unaffected (*n* = 46)
*CYP3A4*	chr7:99361620	ND	NA	Missense	A	c.884T>C	0	1	0
*CYP27A1*	chr2:219677022	rs2229381	0.001	Missense	C	c.524C>T	1	0	4
*LRP2*	Chr2:169985338	rs34564141	0.007	Missense	C	c.13803G>A	2	1	1
*LRP2*	Chr2:169997025	rs764880181	NA	frameshiftDeletion	TG	c.13139delC	1	0	0
*LRP2*	Chr2:170003432	rs4667591	0.712	Missense	T	c.12628A>C	35	12	45
*LRP2*	Chr2:170010985	rs2075252	0.763	Missense	T	c.12280A>G	32	13	45
*LRP2*	Chr2:170013904	rs79723119	0.008	Missense	A	c.11996T>G	1	0	1
*LRP2*	Chr2:170029657	rs34355135	0.006	Missense	C	c.11092G>A	2	0	0
*LRP2*	Chr2:170038761	rs3213760	0.003	Missense	C	c.9914G>A	1	1	0
*LRP2*	Chr2:170053505	rs2228171	0.267	Missense	C	c.8614G>A	6	3	8
*LRP2*	Chr2:170060603	rs17848169	0.029	Missense	T	c.7894A>G	2	4	7
*LRP2*	Chr2:170062977	rs61995915	0.013	Missense	T	c.7253A>G	2	2	3
*LRP2*	chr2:170063250	rs886055084	NA	Missense	G	c.6980C>T	0	0	2
*LRP2*	chr2:170070172	rs4667596	0.024	Missense	C	c.6035G>A	1	0	1
*LRP2*	chr2:170097707	rs17848149	0.030	Missense	T	c.3836A>C	1	0	2
*LRP2*	chr2:170113670	rs150752263	0.001	Missense	G	c.2603C>G	2	0	1
*LRP2*	Chr2:170129547	rs34291900	0.028	Missense	C	c.2006G>A	2	4	7
*LRP2*	chr2:170136882	ND	NA	frameshiftDeletion	CA	c.1318delT	1	0	0
*LRP2*	Chr2:170147502	rs34693334	0.063	Missense	C	c.775G>C	2	0	4
*LRP2*	Chr2:170175334	rs2229263	0.278	Missense	T	c.248A>G	19	7	19
*CUBN*	chr10:16870912	rs1801232	0.086	Missense	G	c.10656C>A	5	5	10
*CUBN*	chr10:16877080	rs7898873	0.023	Missense	G	c.10295C>G	1	1	1
*CUBN*	chr10:16911671	rs148491916	<0.001	Missense	C	c.9418G>A	0	0	1
*CUBN*	chr10:16918947	ND	NA	frameshiftDeletion	AG	c.9054_9054delC	2	0	2
*CUBN*	chr10:16918997	rs1801240	0.105	Missense	T	c.9005A>G	8	6	13
*CUBN*	chr10:16919052	rs1801239	0.087	Missense	T	c.8950A>G	7	5	12
*CUBN*	chr10:16930419	rs45569534	0.014	Missense	C	c.8902G>C	1	0	0
*CUBN*	chr10:16932490	rs1801238	0.028	Missense	G	c.8635C>A	3	0	0
*CUBN*	chr10:16942818	ND	NA	Missense	T	c.8216A>G	1	0	2
*CUBN*	chr10:16943371	rs2796835	1.000	Missense	G	c.8150C>G	35	12	46
*CUBN*	chr10:16948277	rs144626884	<0.001	Missense	T	c.7837A>C	1	0	2
*CUBN*	chr10:16948390	rs3740168	0.041	Missense	G	c.7724C>G	1	0	4
*CUBN*	chr10:16961995	rs2271460	0.016	Missense	A	c.6788T>G	0	0	2
*CUBN*	chr10:16962122	rs143291127	<0.001	Missense	C	c.6661G>A	0	0	2
*CUBN*	chr10:16967362	ND	NA	Missense	A	c.6524T>G	2	1	3
*CUBN*	chr10:16967401	rs1276712	0.994	Missense	C	c.6485G>A	35	13	46
*CUBN*	chr10:16967586	rs62619939	0.129	Missense	C	c.6459G>C	7	2	9
*CUBN*	chr10:16979606	rs2356590	0.063	Missense	G	c.5911C>A	1	0	1
*CUBN*	chr10:16979714	rs41289305	0.144	Missense	T	c.5803A>G	6	2	12
*CUBN*	chr10:16982061	rs2271462	0.075	Missense	C	c.5518G>A	1	0	1
*CUBN*	chr10:16989271	rs74116778	0.017	Missense	C	c.5305G>A	1	1	0
*CUBN*	chr10:17024503	rs1801231	0.767	Missense	G	c.4675C>T	34	13	44
*CUBN*	chr10:17110639	rs148869805	0.004	Missense	T	c.2756A>G	2	0	4
*CUBN*	chr10:17113456	rs138083522	0.007	Missense	C	c.2594G>A	1	1	1
*CUBN*	chr10:17126383	rs7905349	0.028	Missense	G	c.2188C>T	0	0	1
*CUBN*	chr10:17147521	rs1801224	0.613	Missense	G	c.1165C>A	29	12	37
*CUBN*	chr10:17153023	rs78201384	0.004	Missense	C	c.910G>A	2	1	2
*CUBN*	chr10:17156151	rs1801222	0.727	Missense	A	c.758T>C	34	13	44
*CUBN*	chr10:17171176	rs12259370	0.007	Missense	C	c.196G>A	1	1	0
*CYP24A1*	chr20:52774635	rs6068812	<0.001	Missense	A	c.1226T>C	2	0	1
*VDR*	chr12:48272895	rs2228570	0.631	Missense	A	c.152T>C	30	12	41
*PDIA3* *PDIA3*	chr15:44055344 chr15:44038766	rs139812953 ND	<0.001 NA	Missense Missense	A C	c.542A>G c.29C>T	1 0	1 0	2 2
*RXRA*	chr9:137309155	rs61751479	0.002	Missense	G	c.762G>A	0	0	3
*GC*	chr4:72618296	rs9016	0.998	Missense	T	c.1391A>G	35	13	46
*GC*	chr4:72618311	ND	NA	Missense	T	c.1376A>G	1	0	0
*GC*	chr4:72618323	rs4588	0.250	Missense	G	c.1364C>A	22	9	25
*GC*	chr4:72618334	rs7041	0.515	Missense	A	c.1353T>G	31	10	41
*GC*	chr4:72669661	rs76781122	0.018	Missense	C	c.3G>T	2	0	4
*FCGR2A*	chr1:161476204	rs201218628	NA	Missense	CA	c.187_188delCAinsTG	8	3	12
*FCGR2A*	chr1:161479745	rs1801274	0.478	Missense	A	c.500A>G	32	10	40
*FCGR2C*	chr1:161559571	rs138747765	0.279	Missense	C	c.353C>T	12	6	17
*FCGR2C*	chr1:161561156	rs76016754	0.010	Missense	A	c.614A>T	2	0	0
*FCGR3A*	chr1:161512873	rs115866423	0.010	Missense	T	c.1009A>T	2	0	3
*FCGR3A*	chr1:161514542	rs396991	0.324	Missense	A	c.841T>G	22	10	26
*FCGR3A*	chr1:161518214	rs148181339	NA	Missense	T	c.631A>G	3	0	4
*FCGR3A*	chr1:161518333	rs10127939	0.054	Missense	A	c.512T>A	3	1	9
*FCGR3A*	chr1:161518333	rs10127939	0.054	Missense	A	c.512T>G	5	3	3
*FCGR3A*	chr1:161518336	rs77144485	0.091	Missense	C	c.509G>A	18	7	24
*FCGR3A*	chr1:161519601	rs770473456	<0.001	Missense	A	c.241T>C	0	1	0
*FCGR3A*	chr1:161519622	rs773823413	<0.001	Missense	C	c.220G>A	0	1	0
*FCGR3B*	chr1:161595986	rs200215055	0.001	Missense	C	c.634G>T	1	1	1
*FCGR3B*	chr1:161599654	rs5030738	0.040	Missense	G	c.341C>A	1	1	2
*FCGR3B*	chr1:161599693	rs448740	0.636	Missense	T	c.302A>G	34	11	40
*METTL21B*	chr12:58174368	rs141172155	0.001	Missense	G	c.620G>A	0	0	2
*FGF23*	chr12:4479549	rs7955866	0.126	Missense	G	c.716C>T	7	1	8
*DAB2*	chr5:39376988	rs3733801	0.133	Missense	C	c.1901G>A	13	4	15
*DAB2*	chr5:39377132	rs700241	0.023	Missense	G	c.1757C>T	0	0	1
*AVIL*	chr12:58204283	rs2172521	1.000	Missense	T	c.610A>G	35	13	46
*AVIL*	chr12:58209772	rs753181730	<0.001	Missense	C	c.52G>A	1	0	1

AID, autoimmune disease; ND, no data; NA, not available. Genes involved in the regulatory pathway of *CYP27B1* gene have also been added at the end of this table.

### 3.3 Prioritization of variants

**Figure 3:**
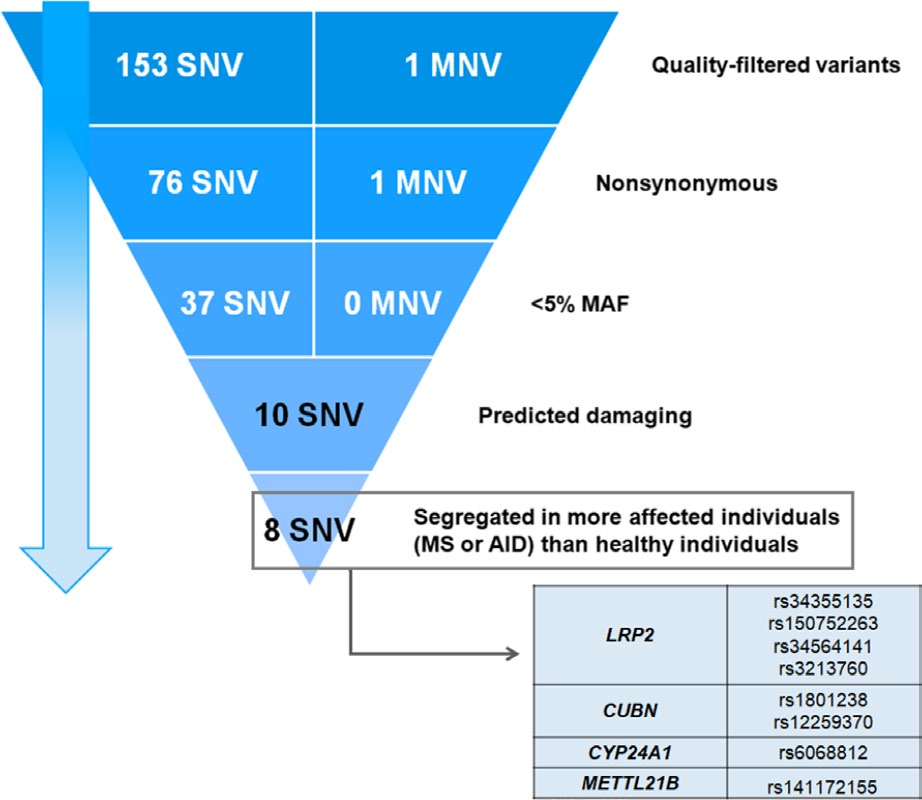
Prioritization of variants of genes involved in the vitamin D signaling pathway. SNV, single nucleotide variants; MNV, multiple nucleotide variants, repeats two or more SNV in successions

Figure 3 illustrates the filtering and prioritization process. Prioritization yielded eight exonic variants meeting the established criteria, located on the genes *LRP2* (four variants), *CUBN *(two variants), *CYP24A1 *(one variant), and *METTL21B *(one variant).

### 3.4 Variants affecting levels of circulating vitamin D

In the families, we detected single variants of *CYP3A4*,* CYP27A1*,* CYP24A1*, and five variants of the *GC *gene, with no significant differences between groups. We also analyzed the genes that regulate *CYP27B1* expression (*METTL21B*, *METTL1*, *FGF23*, and *PTH*) (Table 1). Seven patients with MS (20%), one with an AID other than MS (7.7%), and eight unaffected individuals (17.4%) were carriers of *FGF23* variant rs7955866. Regarding family type, this variant was present in six members of type‐A families (13.6%) and 10 members of type‐B families (29%). *METTL21B* variant rs141172155 was found in two patients with MS (5.7%) and in no other group. We observed two additional synonymous variants of the genes *METTL21B* (rs923829) and *PTH* (rs6256). Variant rs923829 was more frequent in type‐A than in type‐B families (16 [36.4%] vs. 8 [16%]). After prioritization and filtering, we detected rs6068812 variant of *CYP24A1*in one patient with RRMS, one with PPMS, and one unaffected individual from a type‐B family. We analyzed the variants from genes potentially involved in the 12q LD block (*CDK4*, *TSFM*, *AGAP2*, *AVIL*, *CYP27B1*, *METTL1*, and *METTL21B*) and compared these against variant from *CYP24A1 *(rs6068812). All three individuals with rs6068812 also had a variant in *AVIL *gene (rs2172521), which was present in all members of our cohort. Therefore, no association was observed between the 12q LD block genes variants analyzed and *CYP24A1 *gene variant (rs6068812). None of these variants found in the families had a significant relationship to MS or MS with AID compared to unaffected family members.

### 3.5 Variants affecting vitamin D internalization

We observed 18 nonsynonymous variants of *LRP2*, none of which was associated with MS. We also detected 29 variants of *CUBN*. The majority of variants observed after filtering and prioritization affected VD internalization. *LRP2* variant rs34564141 was observed in two patients with RRMS associated with another AID (ulcerous colitis and Guillain‐Barré syndrome), one individual with autoimmune uveitis, and one unaffected individual from a type‐B family. *LRP2* variant rs34355135 was detected in two individuals with RRMS, one of whom also had autoimmune uveitis, from a type‐B family. *LRP2* variant rs3213760 was identified in one patient with RRMS and one family member with hypothyroidism from a type‐B family. *LRP2* variant rs150752263 was observed in two patients with RRMS and an unaffected individual from a type‐A family. Regarding the *CUBN* gene, variant rs1801238 was identified in two patients with RRMS and one with PPMS (from tow type‐B families); two of these patients (siblings) had an additional AID (hypothyroidism and systemic lupus erythematosus). Variant rs12259370 was observed in one individual with RRMS and comorbid rheumatic fever and one individual with hypothyroidism from a type‐B family. We also identified a family in which the variants rs3213760 (*LRP2*) and rs12259370 (*CUBN*) were present in an individual with hypothyroidism and in no patients with MS. We found two nonsynonymous variants of *DAB2* (rs3733801 and rs700241), which codes for a protein involved in VD endocytosis. No differences were detected between groups (MS, other AIDs, or unaffected individuals; type‐A or type‐B families). Variant rs3733801 was observed in 13 patients with MS (37.1%), four individuals with other AIDs (30.8%), and 15 unaffected individuals (32.6%). Regarding family type, this variant was observed in 12 members of type‐A families (27.3%) and 20 members of type‐B families (40%). Variant rs700241 was detected in one unaffected individual from a type‐B family (Table 1). None of these variants found in the families had a significant relationship to MS or MS with AID compared to unaffected family members.

### 3.6 *VDR* and *RXRA* variants

We observed only one variant of *VDR* and one variant (rs61751479) of *RXRA *(Table 1). The *VDR *variants observed were *TaqI* (rs731236), in 30 patients with MS (85.7%), 11 individuals with other AIDs (84.6%), and in 35 unaffected individuals (76.1%); and *FokI *(rs2228570) in 30 patients with MS (85.7%), 12 individuals with other AIDs (85.7%), and in 41 unaffected individuals (89.1%). Nine patients with MS (25.7%), three patients with other AIDs (23.1%), and 12 unaffected individuals (26.1%) were homozygous for the G allele of *TaqI*. GG homozygosity for *FokI* was observed in 17 patients with MS (48.6%), five patients with other AIDs (38.5%), and 15 unaffected individuals (32.6%). Analysis of the differences between family types for these variants revealed that *TaqI* (rs731236) was present in 43 members of type‐A families (97.7%) and 33 members of type‐B families (66.0%). No significant differences between family types were observed for *FokI* (rs2228570) (41 members of type‐A families [93.2%] vs. 42 members of type‐B families [84.0%]). None of these variants found in the families had to do with unaffected family members.

### 3.7 *PDIA3* variants

Two missense variants have been detected on the gene encoding *PDIA3* in our cohort. One of them, rs139812953, is only observed in a MS case, in a case with other AID and in two unaffected cases. The second one, positioned in chr15:44038766 is only present in two nonaffections.

## 4 DISCUSSION

Several authors have hypothesized that predisposition to MS and other AIDs may involve VD signaling pathway components in which various potentially associated variants have been described. This theory is supported by some case‐control studies which have analyzed variants at different points of the pathway; results are conflicting, however, with some studies finding no association (Agnello et al., 2017; Simon, Munger, Yang, & Ascherio, 2010). This hypothesis involves two possible underlying mechanisms: firstly, genomic variations may reduce the effectiveness of VDR function; and secondly, these variations may cause a change in gene expression in either immune or CNS cells (Lu, Taylor, & Körner, 2018). The most complex issue in the study of the VD metabolic pathway is related to the heterogeneity of the pathway depending on the cell or tissue type. Accounting for the fact that different cell types internalize VD in different ways, our WES study of the different variants in the families of patients with familial MS included variations in all subpathways of the VD signaling pathway. VDR action is also variable after translocation to the nucleus, depending on the target cell (Zella, Kim, Shevde, & Pike, 2006; Zella et al., 2010). It has been suggested that this is due to different levels of local activators and coregulators and epigenetic mechanisms (Saccone, Asani, & Bornman, 2015); this issue lies beyond the scope of the present study. The influence of specific tissue or cell type prevents us from easily drawing conclusions as to the potential role of VD based only on levels of some of its metabolites in the blood.

### 4.1 Genes influencing circulating vitamin D

Some variants had been described in the literature in association with MS that may act through an effect on circulating VD (Alloza et al., 2012; Australia New Zealand Multiple Sclerosis Genetics Consortium, 2009; Cortes et al., 2013; Karaky et al., 2016; Laursen et al., 2015; Manousaki et al., 2017; Orton et al., 2011; Ramagopalan et al., 2011; Ramasamy et al., 2014; Ross et al., 2014; Scazzone et al., 2018; Simon et al., 2011; Sundqvist et al., 2010; Zhuang et al., 2015). No significant intergroup differences were observed for presence of these variants in our cohort. The results obtained are shown in Table S1. We did locate the *CYP24A1* variant rs6068812 in one patient with RRMS, one with PPMS, and one unaffected individual from a type‐B family; this variant has not been associated with MS in the literature. Given the very low MAF of the variant, we should consider a potential association with the disease and could be analyzed in further studies.

This stage of the VD metabolic pathway is one of those in which researchers have searched for variants influencing MS, especially in *CYP27B1*; it is therefore surprising that no association was found. Associations have been described between VD deficiency and numerous genetic variants (Lafi, Irshaid, El‐Khateeb, Ajlouni, & Hyassat, 2015; Li et al., 2014; Lu et al., 2012; Nissen et al., 2014; Signorello et al., 2011; Slater, Rager, Havrda, & Harralson, 2017; Wang et al., 2010; Zhang et al., 2012) (Table S1). However, these associations do not necessarily represent increased risk of MS, as no correlation has been demonstrated between the disease and plasma VD level (Ahn et al., 2010). This is probably due to the existence of confounding factors (Bu et al., 2010), including geographical (Elkum et al., 2014) or ethnic (Batai et al., 2014) variation, as well as environmental factors influencing plasma VD levels, such as sex, age, the season in which samples are taken (Engelman et al., 2013), the use of dietary vitamin D supplements, consumption of milk and cereals, obesity, and daily amount of time spent walking (Hansen et al., 2015). The biomarker used also represents a methodological bias that may affect study results: 25(OH)D and 1,25(OH)_2_D have been associated with different findings (Engelman et al., 2008). Regarding the regulation of *CYP27B1* expression (genes *METTL21B*, *METTL1*, *FGF23*, and *PTH*), which is thought to influence *VDR *(Bouksila et al., 2018), *METTL21B* variant rs141172155 was observed only in two patients with MS from type‐B families. We did not detect any *METTL1* variant associated with MS (Alcina et al., 2013). This pathway is of interest, as *CYP27B1 *may be influenced by processes occurring in the kidneys in MS and other AIDs (Meyer et al., 2017). GWAS data suggest that there may be a specific locus for MS in a LD block affecting 17 genes on the long arm of chromosome 12. This area as a whole may therefore be considered a risk locus. However, we did not observe related variants in our analysis. Only two exonic and nonsynonymous variants (rs2172521, rs753181730) belonging to the *AVIL* gene were found in the families. Neither did we observe the proposed relationship with *CYP24A1*, located on chromosome 20 (Gandhi et al., 2010).

### 4.2 Genes influencing the cellular internalization of 1,25(OH)_2_D

Uptake of the 25(OH)D‐DBP complex by the proximal tubules of the kidneys and by other cell types occurs by means of LRP2‐, CUBN‐, or DAB2‐mediated endocytosis; this process maintains serum VD concentrations and activates 1,25(OH)_2_D. Following internalization, DBP is degraded in the lysosomes, releasing 25(OH)D, which is activated by CYP27B1 to 1,25(OH)_2_D. Therefore, functional alterations in LRP2, CUBN, or DAB2 could lead to excessive urinary excretion of the 25(OH)D‐DBP complex and reduced activation of 1,25(OH)_2_D by VDR. In this way, VD internalization can be mediated by LRP2, with or without the participation of CUBN and the DAB2 adaptor protein, although vitamin D also accesses some cells freely. Zhou et al. (2017) describe an association between MS relapses and *LRP2* variant rs12988804. The study was based on data from three cohorts of patients with MS, including one series of paediatric patients. Variant rs12988804 is located in the intronic region of transcript NM_004525.2, as well as in the transcript variants XM_011511183.2 and XM_011511184.2. Because intronic variants do not participate in protein synthesis, they rarely have any impact on disease; however, they are occasionally involved in pathophysiology due to their influence on splicing regions. Variant rs12988804 was not detected in our cohort. While it is a frequent variant (MAF 0.206), it is located in an intron, whereas our study focused on exonic regions. Zhou et al. signal the proximity to variant rs754235034 as the potential mechanism by which rs12988804 is associated with MS. We identified four exonic variants: rs34564141, rs34355135, and rs3213760 in type‐B families and rs150752263 in a type‐A family. All four were detected in patients with AIDs other than MS; it could therefore be the case that variants of *LRP2* are associated with AIDs in general, rather than MS specifically. This is consistent with our hypothesis about the effect of family type. Similarly, we observed AID‐associated *CUBN* variants (rs1801238 and rs12259370) in type‐B families; one type‐B family included a patient with a non‐MS AID who displayed variants of both *LRP2* (rs3213760) and *CUBN *(rs12259370). We also analyzed variants of *DAB2*, which, together with *LRP2 *and *CUBN*, is involved in vitamin D internalization. Variant rs3733801 was the most frequent, although no difference was observed in its frequency between patients with MS and the other individuals studied; it was more frequent in type‐B than in type‐A families, however. This could support the hypothesis that these families have more variants of genes related to VD internalization, which could represent a genetic predisposition to AIDs and could be analyzed in further studies.

### 4.3 *VDR *and *RXRA *genes variants

Vitamin D receptor variants are one of the genetic biomarkers which have aroused the most interest in evaluating the risk of AID. They have been associated with increased risk of a range of AIDs, including systemic lupus erythematosus (Carvalho et al., 2015), rheumatoid arthritis (Cavalcanti et al., 2016), autoimmune thyroid disease (Feng, Li, Chen, & Zhang, 2013), and ankylosing spondylitis (Cai et al., 2016). The most studied *VDR *variants are *BsmI*, *FokI*, *ApaI,* and *TaqI*, although other variants have been described (Dickinson et al., 2009). The *ApaI*and *BsmI* variants are located close to the 3′ end of the *VDR *gene, in the intron between exons 8 and 9, and cannot be analyzed in WES studies. The *FokI* (rs2228570) and *TaqI* (rs731236) variants, on the other hand, can be detected with this technique. The findings published in the literature are conflicting, and these variants have been related with both risk and progression of MS (Altemaimi, Alenezi, Alserri, Alroughani, & Al‐Mulla, 2015; Čierny et al., 2016; Fukazawa et al., 1999); other studies observe no association, however. Meta‐analyses have not solved this controversy, finding both negative (García Martín et al., 2013; Huang & Xie, 2012) and positive results (Tizaoui, Kaabachi, Hamzaoui, & Hamzaoui, 2015), although they do suggest that ethnicity, age, and geographic latitude may influence the associations between these variants and MS risk. We found no significant differences in rs2228570 and rs731236 frequency between patients with MS, patients with other AIDs, and unaffected family members. The only Spanish study into *VDR *variants in sporadic MS did find an association (García Martín et al., 2013). Variant rs731236 was significantly more frequent in members of type‐A than type‐B families, whereas rs2228570 prevalence was similar in both groups. No associations were detected for variants of the gene encoding RXRA, a transcription factor that together with VDR forms heterodimers needed for nuclear translation, similar to the findings of a recent study (Agnello et al., 2018).

### 4.4 *PDIA3 *gene variants

Protein disulfide isomerase family member 3 was recently described as a VD receptor (Doroudi, Olivares‐Navarrete, Boyan, & Schwartz, 2015). Considering that its presence in the brain is greater than VDR, it has been suggested that would have a greater role in the CNS disorders (Tohda, Urano, Umezaki, Nemere, & Kuboyama, 2012) since it is present in practically all cell types and acts in nongenomic functions of the VD. There is no previous information on the influence of variants on the *PDIA3 *gene in MS (Landel, Sthefan, Cui, Eyles, & Feron, 2018). We observed only two variants of *PDIA3 *gene in our cohort but no associations were observed in MS or AID groups. However, information on the signaling pathway of *PDIA3* is scarce, so further studies will probably be needed.

### 4.5 Analysis of variants in families

The hypothesis of genetic regulation of vitamin D in families has been proposed in studies of twins, which observed that certain variants, particularly of *CYP27B1*, may influence the risk of MS (Orton et al., 2008); however, very little information is available on this subject. A *CYP27A1* mutation thought to be related to MS has been described in a family including three patients with the disease (Traboulsee et al., 2017). Another published study design, using WES to study father‐mother‐child trio, includes 28 patients from eight families; however, the results are not known (García‐Rosa et al., 2017). The only published study on the subject, evaluating *VDR* variants in 29 patients with familial MS and comparing them to unrelated controls, found a significant difference between groups for the *TaqI* variant of *VDR *(Yucel et al., 2018). Our study is the first to analyze whole families and to compare patients to family members, thereby eliminating potential confounders. Furthermore, families were divided into the two types described above (A and B) according to the generational distribution of cases. Analysis of the variants detected in the different pedigrees demonstrates the presence in some families of variants which may appear to be linked to the disease, although this was not supported by the results of the comparative analysis of the entire cohort. Given the variants detected, we may hypothesize that type‐B families, with a probable greater genetic load and greater prevalence of other AIDs, are the best group to study in order to analyze the role of the genetic variants in each family. In a type‐B family, one variant in *FCGR2C *gene (rs76016754) was observed in homozygosis in an MS case, but not in other MS patients. Due to that, further cases will be necessary to clarify its significance.

### 4.6 Study limitations

Genetic factors have a clear influence over susceptibility to MS. Linkage analysis using single‐nucleotide variants, and especially GWAS studies, have made it possible to detect loci related to the disease. However, the alleles detected are neither necessary nor sufficient to cause MS; GWAS may also be unable to detect rare variants that may have a significant effect. While GWAS is based on the hypothesis of common variants related to the disease, and provides information on the risk associated with common genetic variability (Simón‐Sánchez & Singleton, 2008), WES is based around the idea of risk associated with rare variants, such as *TYK2* variant rs55762744, which was identified with this technique (Dyment et al., 2012). Evidently, we are not restricted to the use of a single one of these approaches to research the genetic basis of MS (Jiang, Tan, Tan, & Yu, 2014); rather, they are mutually enriching.

WES has a number of disadvantages. The first of these is incomplete coverage: it does not encompass the whole genome, and yields a high number of rare variants of uncertain importance; we should also mention the ethical dilemma involved in the unexpected discovery of incidental findings of variants associated with diseases that were not the target of the study (Klein & Foroud, 2017). Secondly, identifying multiple rare variants within a single gene makes it difficult to establish a clear interpretation of the gene's role. Finally, there is a high probability of false positives due to the large quantity of sequencing data generated and the unclear likelihood of causal relationships. GWAS also has the disadvantage of detecting single‐nucleotide variants that may be associated with disease but have no known function related to the disease. This may mean that the true causal gene is a nearby, rare variant. Nevertheless, this limitation is reduced if the full genome or exome is sequenced; analysis aims to establish how the variant is related to the disease.

Familial studies search for genetic factors from different perspectives than studies of sporadic cases of MS. However, these study types cannot replace one another; rather, the data they yield are complementary. These studies offer the advantage of controls being members of the same family, preventing such confounding factors as ethnic and often geographical differences. They are also characterized by greater genetic homogeneity between individuals, and enable a better understanding of associated autoimmunity. However, they also involve certain disadvantages, such as small sample sizes (Wang et al., 2016) and reduced statistical power when analysing the cohort as a whole. Analysis of whole families, as performed in the present study rather than trios (comparing patients to first‐degree relatives), increases the power of family studies. This is of special interest in studies where factors may also have a considerable environmental influence, as is the case of vitamin D. Studying cases and controls from a single family reduces biases related to exposure to environmental factors, compared to studies with controls from the general population. However, it may not be possible to extrapolate information from familial forms of MS to patients with sporadic forms.

A limitation of our study is that the unaffected cases were classified according to the data obtained from direct interrogatory, but brain magnetic resonance was not performed. Thus, we cannot exclude the existence of subclinical MS.

As previously discussed, the great difficulty with WES is addressing and interpreting large numbers of variants. Our study was limited to exonic variants, which we can expect to be functional. Although this technique does allow the detection of intronic variants located close to exonic regions, causality is very difficult to interpret, particularly with small sample sizes. For this reason, we do not address some of the associations described in the literature.

## 5 CONCLUSIONS

Multiple S is considered to be a polygenic disease and it is very doubtful that the risk may depend on one or few variants. It is more likely to depend on alterations on signaling pathways genes such as the ones related to VD pathway. In order to analyze the data of the studies, it seems necessary to determine the prevalence of the variants in these pathways in the families of patients with MS and especially in the different forms of MS families. Our study aimed to know which variants appear in families of MS. Table 1 includes the 77 nonsynonymous exonic variants in genes participating in the VD pathway found in the subjects families that present more than one patient with MS and that could be related to VD functionality. The genes with the greatest presence of variants correspond to those that affect the entry mechanisms in the cells, spatially *CUBN* and *LRP2*. However, the presence of all the variants found does not differ between the cases and the not affected members in the family. When analyzing the pedigrees of each family included in the study, a relationship of these variants with MS has not been observed either.

This is the first study to address the whole family of patients with familial MS using WES and focusing their analysis into the VD metabolic pathway as a whole, analyzing the potential genetic influences over the pathway. Despite the number of studies reporting an association between genetic variants affecting the VD signaling pathway, MS and other AIDs, the information obtained is relatively limited. After comparison with unaffected family members and analyzing the pedigrees, the majority of the variants published appear not to be specific to MS. This might be due to the fact that sporadic and familial forms involve different genetic factors, or that GWAS led to false positives in comparisons between patients and controls. Our study did not reproduce the association of MS risk with a *CYP27B1 *variant, or with exonic variants of *VDR*. We did detect several variants of interest in *LRP2* and *CUBN*, as well as *CYP24A1 *and *METTL21B *variants; these should be confirmed by further studies. We have not observed any relationship between variants in the gene that encodes the PDIA3 receptor with the studied groups.

Furthermore, we did not identify any relationship with genetic variants previously associated with MS, including in type‐B families, which we had hypothesized would display a greater influence due to genetic load; and there are two potential explanations for this. First, familial MS may not be directly influenced by the genetic alterations that affect VD signaling pathways in sporadic MS, which are detectable by GWAS. The second possible explanation is that vitamin D may be an environmental factor that influences the risk of MS but is not determined by genetic variants. This theory is supported by the variability of the cells and tissues in which the VD metabolic pathway occurs, and the fact that the pathway is influenced by local molecular and epigenetic factors, which makes it difficult for epidemiological and intervention studies to understand (uncover) VD's possible role in the disease. Although this study does not allow us to confirm either hypothesis, it indeed provides information on the variants found in the VD signaling pathway in MS families.

## CONFLICTS OF INTEREST

The authors have no conflicts of interest to declare.

## ETHICS AND CONSENT

This study was approved by the Clinical Research Ethics Committee of *Hospital Clínico San Carlos*. All participants included in the study signing a written consent. Data were handled in observance of the Spanish legislation regarding data protection (Organic Law 15/1999 of 13 December). The project was carried out in accordance with the principles included in the Declaration of Helsinki (Recommendations Guiding Physicians in Biomedical Research Involving Human Subjects, Helsinki 1964, amended October 2013).

## AUTHOR CONTRIBUTIONS

Lead researcher: JMG; study design: VP, JAMG, SA, JMG; patient assessments: JAMG, PME, JMG; family studies: VP, LTF, PME; coordination of information: VP, LTF, UGP; WES: SA, JBR, PM; database: VP, JAMG, LTF, JMG; data filtering and analysis: VP, LTF, SA, JBR, PM, JMG, JAMG; statistical analysis: VP, UGP, JMG, JAMG; analysis of results: VP, LTF, JMG, UGP, JAMG; figures and tables: VP, LTF, JMG; manuscript draft: JMG, VP, JAMG, LTF; revision of manuscript; all authors.
