# Supplementary material for: Exonic variants of genes related to the vitamin D signaling pathway in the families of familial multiple sclerosis using whole‐exome next generation sequencing
Source: Brain Behav. 2019 Mar 21;9(4):e01272. doi: 10.1002/brb3.1272 (PMC6456803; doi:10.1002/brb3.1272)
Supplement: Supplementary file 1 [file BRB3-9-e01272-s001.docx]

***Supplementary material table 1.*** Variants of vitamin D pathway genes reported in the literature related to multiple sclerosis.

| **Genes** | **Locus** | **Variant (rs)** | **Location** | **Variant effect** | **Reference allele** | **Change** | **MAF** | **MS** | **Other**  **AID** | **Not**  **sick** |
| --- | --- | --- | --- | --- | --- | --- | --- | --- | --- | --- |
|  |  |  |  |  |  |  |  | **35** | **13** | **46** |
| ***DHCR7*** | chr11:71167449 | rs12785878 | intronic | - | G | c.146+1233G>A | <0.001 | - | - | - |
|  |  |  |  |  |  | c.146+1233G>T | 0.544 |  |  |  |
| ***CYP2R1*** | chr11:14921880 | rs10766197 | nearGene-5 | - | G | - | 0.352 | - | - | - |
|  | chr11:14900931 | rs117913124 | exonic | Synonymous | C | c.1059C>T | 0.017 | 1 | 0 | 4 |
|  | chr11:14914878 | rs10741657 | nearGene-5 | - | T | c.-1127T>C | 0.638 | - | - | - |
|  | chr11:14913575 | rs12794714 | exonic | Synonymous | G | c.177C>T | 0.406 | 20 | 8 | 29 |
| ***GC*** | chr4:72618334 | rs7041 | exonic | Missense | T | c.1353T>A | <0.001 | 31 | 10 | 41 |
|  |  |  |  |  |  | c.1353T>G | 0.515 |  |  |  |
|  | chr4:72608383 | rs2282679 | intronic | - | A | c.*26-796A>C | 0.218 | - | - | - |
|  | chr4:72618323 | rs4588 | exonic | Missense | G | c.1364C>A | 0.250 | 22 | 9 | 25 |
| ***CYP3A4*** |  | NV |  |  |  |  |  |  |  |  |
| ***CYP27A1*** |  | NV |  |  |  |  |  |  |  |  |
| ***CYP27B1*** | chr12:58162739 | rs703842^ | nearGene-5 | Missense | T | c.-1915T>C | 0.378 | - | - | - |
|  | chr12:58157930 | rs118204009 | exonic | Missense | G | c.1166G>A | <0.001 | - | - | - |
| ***RXRA*** |  | NV |  |  |  |  |  |  |  |  |
| ***VDR*** | chr12:48238757 | rs731236 | exonic | Synonymous | T | c.1206T>C | 0.264 | 30 | 11 | 35 |
|  | chr12:48239835 | rs1544410 | intronic | - | G | c.1174+283G>A | 0.337 | - | - | - |
|  |  |  |  |  |  | c.1174+283G>C | NA |  |  |  |
|  |  |  |  |  |  | c.1174+283G>T | NA |  |  |  |
|  | chr12:48238837 | rs7975232 | intronic | - | G | c.1175-49G>T | 0.515 | - | - | - |
|  | chr12:48272895 | rs2228570 (rs10735810) | exonic | Missense | T | c.152T>C | 0.631 | 30 | 12 | 41 |
|  | chr12:48302545 | rs11568820 | nearGene-5 | - | G | NA | 0.363 | - | - | - |
|  | chr12:48298902 | rs11574010 | nearGene-5 | - | G | c.-489G>A | NA | - | - | - |
| ***CYP24A1*** | chr20:52791518 | rs2248359 | nearGene-5 | - | G | c.-1400G>A | 0.458 | - | - | - |
| ***LRP2*** | chr2:170117811 | rs12988804 | intronic | - | G | c.2321-2084G>A | 0.223 | - | - | - |
| ***CUBN*** |  | NV |  |  |  |  |  |  |  |  |
| ***FCGR*** |  | NV |  |  |  |  |  |  |  |  |
| *METTL1^^* | chr12:58165085 | rs10877013 | intronic | - | C | c.111-45G>A | NA | - | - | - |
|  |  |  |  |  |  | c.-1423C>T | 0.375 |  |  |  |
| *METTL21B^^* |  | NV |  |  |  |  |  |  |  |  |
| *FGF23^^* |  | NV |  |  |  |  |  |  |  |  |
| *PTH^^* |  | NV |  |  |  |  |  |  |  |  |

Reference genome: GRCh37.p13 (NCBI)

Reference *NCBI*, *gnomAD*

ND: there is no frecuency data

NV: no described variant

NA: not available

^The SNP rs703842 lies 1.76 kb upstream of *CYP27B1* and in the 3′-untranslated region of the neighbor gene methyltransferase-like protein 1 (METTL1)

^^Genes of the regulatory pathway of CYP24b1 gene (METTL1, METTL21B, FGF23, PTH) have also been added at the end of this table.
